# Designing an Evaluation Battery for Young Male Kayakers: Which Variables to Include? †

**DOI:** 10.3390/sports14080316

**Published:** 2026-07-24

**Authors:** Rui António Fernandes, Fernando Alacid, Beatriz Branquinho Gomes

**Affiliations:** 1 Faculty of Sport Sciences and Physical Education, CIPER (Interdisciplinary Center for the Study of Human Performance), University of Coimbra, 3040-248 Coimbra, Portugal; rui.fernandes@uc.pt; 2 Faculty of Education Sciences, Health Research Center, University of Almeria, 04120 Almeria, Spain; falacid@ual.es; 3 CEMMPRE (Centre for Mechanical Engineering, Materials and Processes), University of Coimbra, 3030-788 Coimbra, Portugal

**Keywords:** physical fitness, young kayakers, performance, equipment, stroke rate

## Abstract

This study aimed to identify the variables that best explain the performance of young male kayakers over 200 and 500 m across two categories, U14 and U16, and to determine whether stroke rate (SR), as described for senior athletes, is also a determining factor in performance in these categories. Twenty-four young Iberian kayakers (Portuguese and Spanish), 13.63 ± 1.50 years, distributed in two competitive categories, U14 (12.67 ± 0.47 years) and U16 (15.57 ± 0.73 years), were evaluated in terms of training, maturity, anthropometry, limb volumes, body composition, equipment, physical fitness, balance, and performance. Water/shoulder distance correlated significantly at both race distances in both age categories. The observed differences between U14 and U16 may reflect the influence of kayaking experience and maturity-related factors, which are likely to affect several performance-related variables. These results suggest the need to produce a specific evaluation battery for U14 that includes the water/shoulder distance, the 20 m shuttle run test, and the BESS test. Another one for U16 consists of the water/shoulder distance test, the pull-up test, and the sit-up test. This study also highlights the importance of designing training programs that enable young kayakers to perform the paddle technique with maximum efficiency at a high SR.

## 1. Introduction

Excellence is the main goal of various art forms and sports. Thus, talent identification implies recognizing, in individuals already involved in sports, the potential to achieve excellence [1]. However, not many theories in sports are as disruptive as the perception of natural talent [2]. As a result, the study of key elements of successful sports is now an important area of interest [3].

Sprint kayaking is held on flatwater courses, and races are contested in nine lanes, at 200, 500, and 1000 m. In kayaking, the trunk rotates from a seated position, with the upper body playing a major role in force production [4]. As a result, kayakers have considerable upper body strength [5]. However, increasing attention has been given to the contribution of the lower limb to paddling technique and performance [6]. Indeed, performance in flatwater kayaking is now recognized as a multifactorial construct arising from the interaction among anthropometric, physiological, biomechanical, neuromuscular, psychological, and nutritional factors, rather than from any single performance attribute [7]. Elite paddlers are typically characterized by high lean body mass, low adiposity, superior aerobic and anaerobic capacities, exceptional upper-body strength and power, efficient paddling technique, and effective neuromuscular coordination, all supported by appropriate psychological preparation and recovery strategies [7].

Quantifying sports performance is difficult once it is described as a multidimensional construct [8] and in the context of youth sports, it is indispensable that studies control for maturity [9]. For example, recent studies with young kayakers have shown that chronological age [10] and biological maturity [11,12] influence performance. However, numerous questions have emerged about the trainability of young athletes, and the answers remain inconclusive. For example, strength is known to increase during childhood and adolescence. However, these variations are attributable to gains in muscle mass and the development of the neuromuscular system. Both prepubertal children and adolescents can demonstrate significant increases in muscle strength with resistance training [13].

Recently, when assessing a group of elite junior, under 23 years of age, and senior kayak paddlers, the bench press one-rep max (1RM) was the best predictor of 200 m kayaking performance [14]. Furthermore, improvements in bench press 1RM have improved kayaking performance. Additionally, measuring physical fitness among children and youth has long been of interest to physical educators. Resistance training in youth has been reported to have several beneficial effects [15]. Gäbler et al. (2021) [10] showed benefits from low-intensity, high-volume strength training regarding the 2000 m performance of young sprint kayakers.

The anthropometric characteristics found in athletes are influenced by the specific constraints of each sport. Many of these traits are caused by both heredity and training, resulting in very different anthropometric profiles. As a result, coaches and researchers have struggled to adapt athletes’ physical and anthropometric profiles to the sport’s specific demands to achieve maximum performance [16]. This is particularly relevant in sprint kayaking, where elite performance has been consistently associated with a mesomorphic physique, high lean body mass, and low body fat, although the relative contribution of these characteristics varies according to age, sex, and competitive level [7]. Similarly, performance may be affected by an incorrect adjustment of the equipment, compounded by discomfort and, subsequently, by an inability to perform the technical movement under optimal conditions [17]. In a study involving young paddlers [18], paddle length showed higher correlations with stretch stature and arm span, suggesting that these measures should be used as criteria for determining paddle dimensions.

Most biomechanical research in sprint kayaking has focused on stroke rate (SR), displacement per stroke (stroke length, SL), and mean boat velocity [19]. Furthermore, previous studies with young kayakers [20,21] have evaluated these variables and included the cycle index (CI), derived from the product of SL and velocity, as an indicator of stroke efficiency [22]. Biomechanical efficiency is considered another major determinant of elite performance, as successful paddlers maximize propulsive force while maintaining stroke efficiency and minimizing hydrodynamic resistance throughout the race [7].

At the senior international level, sprint kayaking performance provides an important benchmark for interpreting the development of young athletes. Because environmental conditions preclude the formal recognition of world records in canoe sprint, the International Canoe Federation (ICF) recognizes World Best Times as the reference standard. The current men’s K1 World Best Times are 33.984 s over 200 m and 97.446 s over 500 m, corresponding to mean boat velocities of approximately 5.89 and 5.13 m·s^−1^, respectively. In championship racing, elite finalists typically complete the K1 200 m in approximately 34.5–36.0 s and the K1 500 m in 98–102 s. These performances are characterized not only by exceptionally high boat velocities but also by the ability to sustain high stroke rates while maintaining stroke length, propulsive impulse, and technical efficiency throughout the race.

In kayaking, boat speed is the product of the SR multiplied by the SL [23]. SR is usually presented as strokes per minute (spm), and an increase in SR is associated with a higher mean kayak velocity and, subsequently, better performance [23,24]. The ability to increase the SR may depend not only on the athlete’s physical preparation but also on paddle dimensions, boat setup, and technique. Elite male paddlers typically achieve stroke rates of approximately 130–160 spm during the 200 m event and 115–130 spm during the 500 m event, indicating that success depends on preserving effective paddling mechanics despite increasing physiological demands. Consequently, these reference values provide an important framework for interpreting youth performance and identifying the technical and physical characteristics that should be progressively developed throughout the athlete pathway.

Therefore, to establish evidence-based assessment batteries for young sprint kayakers, a comprehensive set of anthropometric, maturational, training, physical, balance, equipment, technical, and performance-related variables was systematically evaluated. The aim was to identify the variables that best explain 200 m and 500 m sprint performance in male athletes competing in the Under-14 (U14) and Under-16 (U16) categories, and to determine whether SR, a well-established determinant of performance in adult sprint kayakers, is similarly associated with performance during these developmental stages. By identifying age-specific performance determinants, this study sought to provide an evidence-based framework for developing assessment protocols to support athlete monitoring, training prescription, and long-term talent development.

## 2. Materials and Methods

### 2.1. Participants and Data Collection

This study evaluated 24 young Iberian kayakers (Portuguese and Spanish), aged 13.63 ± 1.50 years, and distributed in two competitive categories, U14 (12.67 ± 0.47 years) and U16 (15.57 ± 0.73 years), assessed in terms of training, maturity, anthropometry, limb volumes, body composition, equipment set-up, physical fitness, balance, and performance. The inclusion criteria were mandatory participation in competitive kayaking for at least 1 year and at least 2 national competitions before the evaluation, approval of the required medical exams, non-smoking, and no alcohol consumption. Additionally, written informed consent from parents or guardians was obtained, and approval of the Ethics Committee (protocol code CE/FCDEF-UC/00322018) for the experimental procedures was obtained.

### 2.2. Procedures

**Figure 1:**
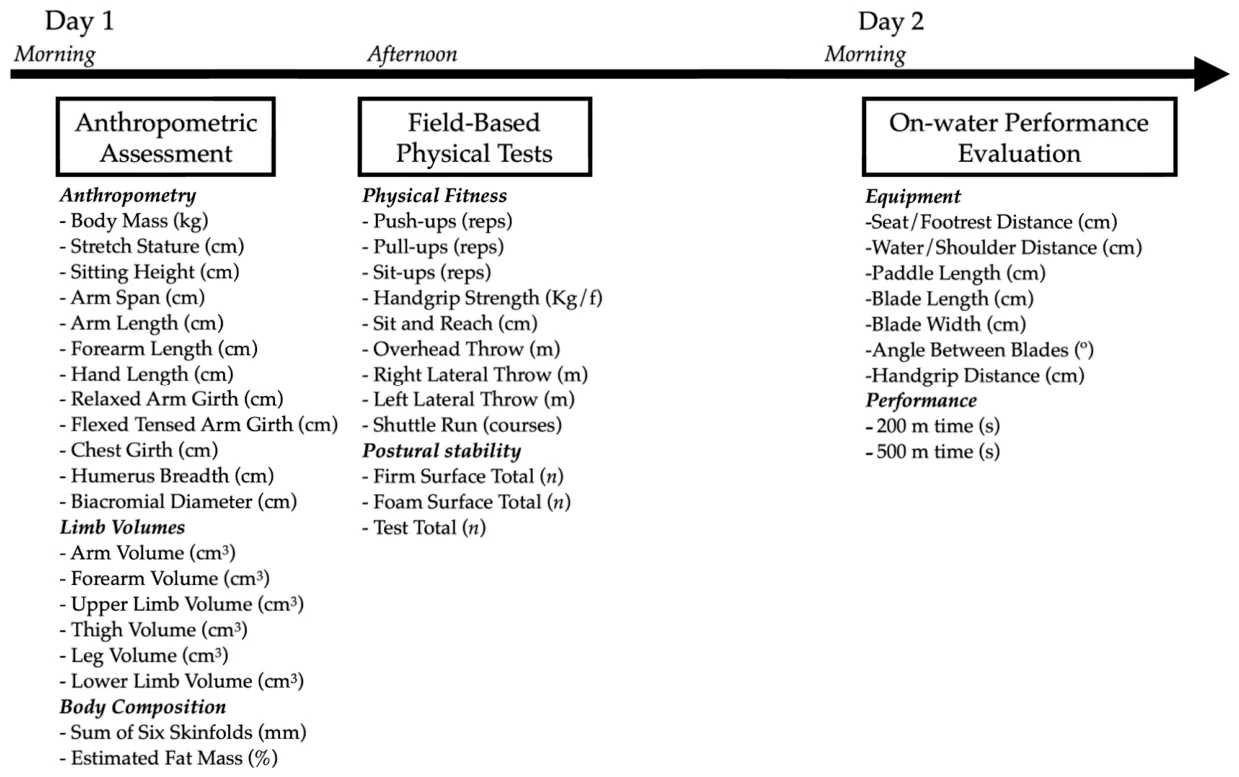
Experimental assessment timeline.

Anthropometric assessments were completed early in the morning, followed by field-based physical tests, and the specific on-water performance evaluation was conducted on the second day, early in the racecourse, under flatwater and fair wind conditions (Figure 1).

### 2.3. Maturity Status

Somatic maturation was used to evaluate biological development [9]. Maturity offset [25] is an indicator of the temporal distribution and estimates the number of years until the subject reaches peak height growth velocity (PHV). This value can be negative (if the subject has not yet reached PHV) or positive (if it has already exceeded PHV). Also, the percentage of predicted adult height (PAH%) was used to define maturity status, and the athlete-predicted adult height (PAH) was estimated using the method.

### 2.4. Anthropometry

All measurements were taken by an experienced researcher following the procedures described by the International Society for the Advancement of Kinanthropometry. Body mass (kg) was measured using a SECA 878 digital scale (SECA, Hamburg, Germany). Stretch stature (cm) and sitting height (cm) were determined with a SECA 206 (Portable stadiometer, SECA, Germany). Arm span (cm) was measured using a metallic tape (Stanley, Towson, MD, USA); the arm, forearm, and hand lengths (cm), humerus, and biacromial breadths (cm) were measured using a sliding caliper (Siber-Hegner GPM Caliper, Bachenbülach, Switzerland). The relaxed arm flexed tensed arm, and chest girths (cm) were measured using an anthropometric tape (Lufkin W606PM, Missouri City, TX, USA).

Skinfold thickness (mm) was measured at six sites (triceps, subscapular, supraspinal, abdominal, front thigh, and medial calf) with a Harpenden skinfold caliper (British Indicators, Burgess Hill, UK), and the percentage of body fat was estimated from the equation defined by [26] using triceps and calf skinfolds.

All instruments were verified and calibrated before each testing session to avoid measurement errors. All measurements were taken two or three times (if the difference between the first two measures was greater than 5% in skinfolds and 1% for the rest of the measures), and the mean or median was used, respectively. The relative technical error of measurement [27] for anthropometric measures ranged between 0.11% and 0.20%.

### 2.5. Limb Volumes

Volumes of the upper and lower extremities were evaluated in the dominant limbs, following the protocol of Rogowski [28] and Jones & Pearson [29], respectively.

### 2.6. Equipment Setup

**Figure 2:**
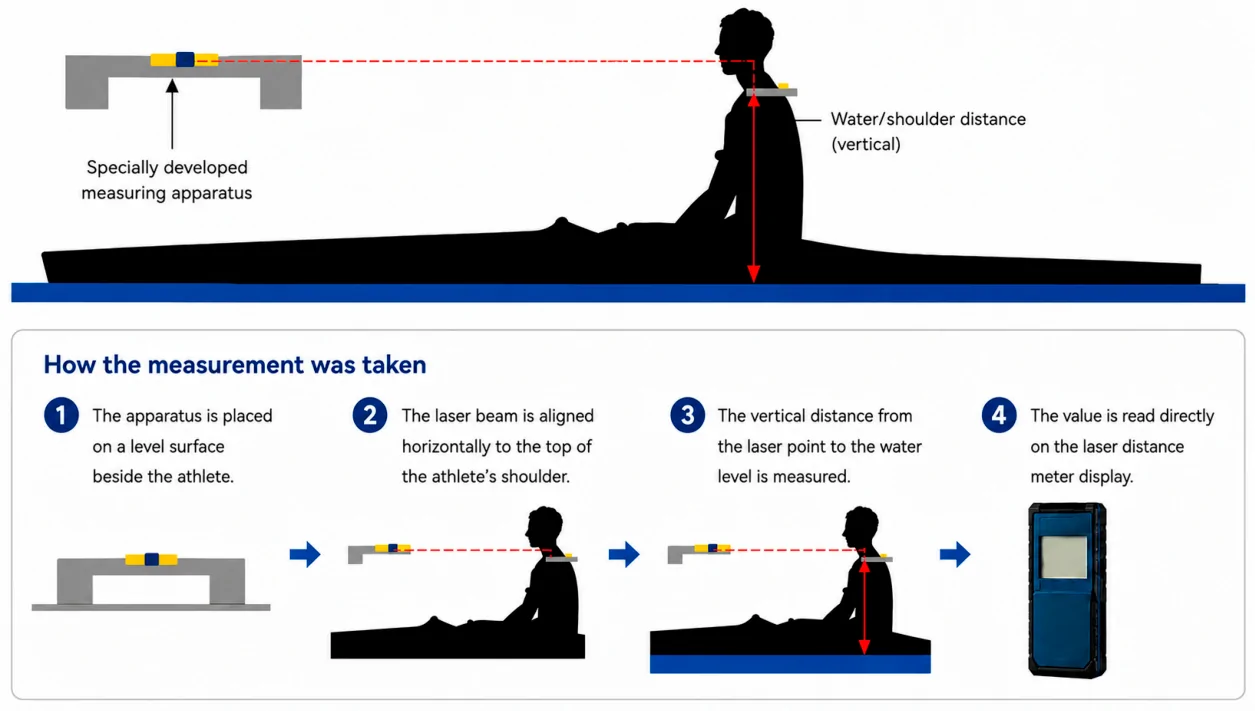
Schematic representation of the procedure for measuring the vertical water–shoulder distance.

For the equipment setup, the variables measured were the paddle and blade length, blade width, handgrip distance, angle between blades, and seat/footrest distance (cm) [30]. In addition, vertical water/shoulder distance (cm) was also included (Figure 2).

Paddle length (cm) was measured with a metal tape (Stanley, Towson, MD, USA). Blade length (cm), blade width (cm), and handgrip distance (cm) were measured using a sliding caliper (Siber-Hegner GPM Anthropometer, Bachenbülach, Switzerland). A self-developed measurement device was used to determine the angle between the paddle blades. The system incorporated a digital inclinometer application (Smart Protractor, version 1.5.8) and was specifically designed to ensure consistent alignment and accurate measurement of blade offset angle. Finally, the vertical water/shoulder distance was measured using a specially developed apparatus and a laser beam (Bosch, Gerlingen-Schillerhöhe, Germany, GLM 40 Professional). All measurements were taken two or three times (if the difference between the first two measures was greater than 1%), and the mean or median was used, respectively.

### 2.7. Physical Fitness

The kayakers’ physical fitness was assessed using an overhead medicine ball throw (3 kg ball) [31]. Given the importance of trunk rotation in the paddle technique [32,33], an adaptation of this test was used for lateral medicine ball throws, with the kayakers adopting a seated position similar to the kayak paddling position. The sit-and-reach test was used to determine hamstring extensibility [34]. The multistage 20 m shuttle run test was used. Pull-up, push-up, and sit-up tests were evaluated following the Fitnessgram test battery [35]. The handgrip strength test was conducted following the Eurofit test battery methodology [36] using a dynamometer (Lafayette Hand Dynamometer, model J00105, Lafayette, IN, USA).

Before all physical tests, the investigators provided clear instructions for each procedure. The general warm-up consisted of eight minutes of multidirectional running activity and five minutes of general dynamic stretching of the upper and lower extremities, delivered and supervised by a certified canoe sprint level 3 coach. During the evaluations, the same test sequence was used. When assessing physical fitness, only the best of three attempts per trial was included in the analysis, with at least 3 min of rest between trials, except for the 20 m multistage shuttle run test, which was performed once. The warm-up consisted of five minutes of familiarization with the materials and procedures used shortly before each test.

### 2.8. Postural Stability

The Balance Error Scoring System (BESS) measures static balance and postural stability. It combines three stances (narrow double-leg stance, single-leg stance, and tandem stance) and uses two footing surfaces (firm surface/floor or medium-density foam). Each stance is held, with hands on hips and eyes closed, for 20 s. For any “Error” for specific actions, including opening eyes, lifting hands off hips, or stepping, stumbling, or falling, points are given, and a higher total score reflects worse performance on the test [37].

### 2.9. Performance

Performance evaluations were conducted in calm water, with no current or significant wind. Participants were asked to complete a 500 m trial, followed by a 60 min interval, and then a 200 m trial. Both trials were carried out in a straight line and in the shortest possible time. Before each test, kayakers were asked to perform their usual pre-race warm-up.

The average SL was calculated using the methodology of Fernandes et al. [23], and the time required to complete both the distance and the SR was obtained with a wireless unit equipped with a 15 Hz GPS and a triaxial accelerometer. This unit contains 12-channel receivers tracking up to 12 satellites, combined with a triaxial accelerometer (GPSPORT, Canberra, Australia) with a sampling rate of 100 Hz, mounted on the stern of the kayak deck. Data were extracted to spreadsheets with Team AMS R1 2016.7 software, then exported to Matlab^®^ R2019b (The MathWorks Inc., Natick, MA, USA) and analyzed using a routine specially developed for this application and already validated [38].

### 2.10. Statistical Analysis

The data distribution and the homogeneity of variance were tested using Shapiro–Wilk and Levene’s tests, respectively. The Spearman correlation coefficient (*r_s_*) was used to determine the relationships between all variables and both performance and SR. Correlations were considered: negligible when 0.3; low when 0.3–0.5; moderate when 0.5–0.7; high when 0.7–0.9; and very high when 0.9–1 [39]. Group mean comparisons for all variables were evaluated using the Kruskal–Wallis test. Also, the ratios of paddle length to handgrip distance (PL:HGD) and sitting height to water/shoulder distance (SH:WSD) were calculated. The level of significance was established at *p* < 0.05. SPSS Statistics 27.0 (SPSS Inc., Chicago, IL, USA) was used for statistical analysis.

## 3. Results

All parameters for the U14 and U16 categories, along with their correlations with performance at 200 and 500 m, are presented in Table 1.

Statistically significant differences were found between U14 and U16 for all variables under study, except APHV, PAH, sum of six skinfolds, percentage of estimated fat mass, angle between blades, and postural stability (firm surface—total, foam surface—total, and test total).

**Figure 3:**
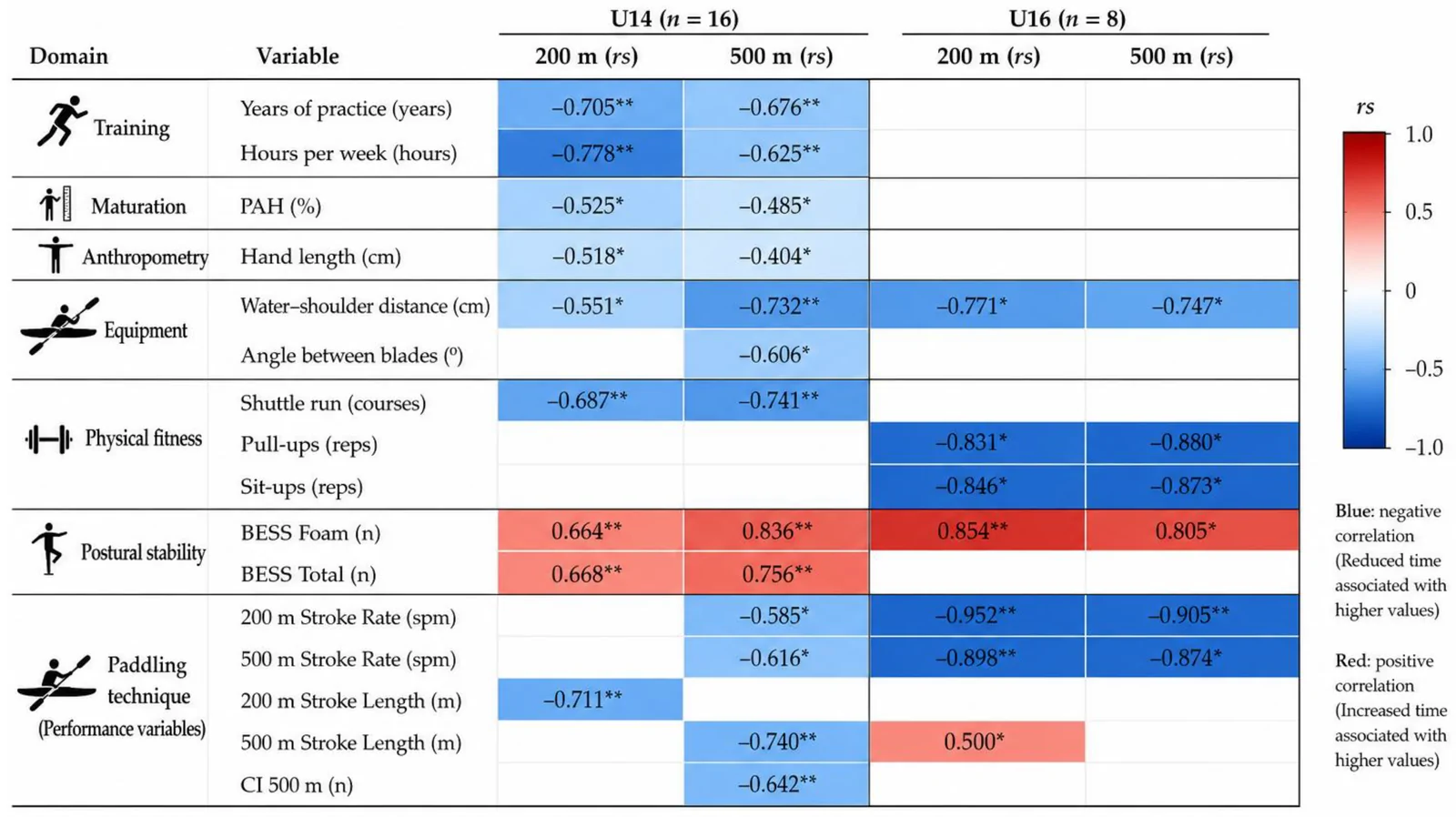
Spearman correlation coefficients (*r_s_*) between performance (200 and 500 m times) and selected variables in U15 and U16 kayakers. Only significant correlations are displayed. * *p* < 0.05; ** *p* < 0.01. Blank cells indicate non-significant correlations (*p* > 0.05). PAH: predicted adult height; CI: cycle index.

In the U14 category, years of practice, weekly training hours, shuttle run performance, BESS foam surface total score, BESS total score, and the CI 500 m were significantly correlated with both 200 m and 500 m performance times. In addition, water–shoulder distance and 500 m SL were significantly associated with performance over both distances. In the U16 category, water–shoulder distance, pull-up performance, and sit-up performance were significantly correlated with both 200 m and 500 m performance times. Furthermore, 200 m SR and the BESS foam surface total score were significantly associated with performance, whereas 500 m SR was significantly correlated with performance over both distances. Figure 3 summarizes the main significant associations found.

Concerning SR, the top three performances at 200 m in U14 and U16 were always above the mean SR (U14: 119.33 ± 3.51 and U16: 132.33 ± 2.51 spm) and the bottom three below the mean SR (U14: 101.00 ± 4.24 and U16: 118.66 ± 3.05 spm; the same could be observed for 500 m, also in both categories, U14: 100.66 ± 2.30 and U16: 109.66 ± 2.51 spm and U14: 94.00 ± 1.73 and U16: 101.00 ± 1.00 spm, respectively.

Table 2 shows the correlation between chronological age (CA), training, maturity, anthropometry, physical fitness, balance, and performance variables with SR and CI at both 200 and 500 m. Regarding training, only in U14 did years of practice correlate significantly with 500 m SR, and hours per week correlated significantly with 200 m SR and 500 m SR. Considering the equipment setup in the U14 category, the water/shoulder distance was significantly correlated with the 500 m SR. The U16 blade width correlated significantly with the 200 m SR and the 500 m SR. For physical fitness, U14 showed significant correlations between shuttle run and 200 m SR and 500 m SR, and U16 showed significant correlations between pull-ups and 200 m SR and 500 m SR, and between sit-ups and 200 m SR and 500 m SR.

Table 3 shows the mean and standard deviation for the PL:HGD and SH:WSD ratios.

The SH:WSD ratio revealed statistically significant differences between U14 and U16. Additionally, the top and bottom U14 200 m performances show SH:WSD ratios below the age-group average (1.77 ± 0.04 and 1.77 ± 0.06 *n*, respectively). Regarding the three top and bottom U14 performances at 500 m, the observed SH:WSD ratio was below average for the top performances (1.77 ± 0.02 *n*) and above average for the bottom (1.84 ± 0.12 *n*). In U16, both the 200 and 500 m performances showed that the SH:WSD ratio was below average for the top performances (1.64 ± 0.06 *n*) and above average for the bottom performances (1.77 ± 0.11 *n*).

Table 4 shows the mean and standard deviation for the sitting height (SH), the water/shoulder distance (WSD), and the SH–WSD difference.

## 4. Discussion

This research aimed to identify the variables that best explain the performance of young male kayakers over 200 and 500 m across two age groups, U14 and U16, to develop more suitable evaluation batteries for each age group. In addition, it aimed to identify whether SR, as described for adult kayakers, is a determining factor in performance in these categories.

The main finding of this study was that only the water/shoulder distance showed a significant correlation across both categories and at both race distances. Furthermore, the differences observed between U14 and U16 kayakers appear to reflect not only the expected age-related differences between categories, but also differences in kayaking experience and biological maturation, which may influence many of the remaining variables assessed. These may reinforce the need to design assessment batteries that accommodate these differences. Also, in accordance with previous studies in adult athletes [23], a higher SR is effectively correlated with performance across the two distances studied and the two age categories.

However, it is essential to note that studying young athletes is complex. Many factors contribute to sporting success, making it challenging for coaches and sports scientists to interpret results, particularly because it is difficult to accurately distinguish in growing athletes whether the results are due to training or maturation.

The CA, anthropometric, and maturational characteristics are intertwined. Gäbler et al. (2021) [10] showed that CA alone may be responsible for the differences observed in the performance of young paddlers. If an athlete is older or more mature than his peers and has been exposed to the growth process earlier and longer, he may have superior anthropometric characteristics. Also, Fernandes et al. (2022) [40] showed that older U16 kayakers born in the first birth quarter performed better than their peers born in other birth quarters, and the fastest kayakers in the U14 category were born in the third quarter of the year.

Furthermore, it seems that those with more years of practice will achieve better performance, regardless of the category they compete in, as shown by Fernandes et al. [11], who stated that young kayakers who performed better were more mature and had more years of kayaking practice. Forbes et al. (2009) [41] have also reported that age, height, and sitting height were all significantly correlated with 1000 m performance. Likewise, [12] evaluated young kayakers’ anthropometric, physical fitness, and specific performance characteristics. They reported significant differences (*p* < 0.05) in body mass, height, sitting height, overhead medicine ball throw, and specific performance tests at 1000, 500, and 200 m by maturity. Gäbler et al. (2021) [10] reported that physical fitness tests show a stronger association with performance when they share biomechanical and physiological similarities with the sport’s demands. In the present study, the right and left lateral medicine ball throws correlated significantly with the 200 m time in the U14 category. In U16, the pull-up test correlated significantly with the time required to perform both distances.

The present study shows that only in the U14 category are maturity indicators significantly correlated with 200 m performance, which may also indicate that kayaking experience serves as a leveling factor among kayakers with different maturity levels, reinforcing the technical nature of the sport and possibly the importance of strength for performance, especially in 200 m.

The significant correlation between the total score of the BESS test and the performances of U14 on both 200 and 500 m, and of U16 on 200 m, supports the idea that, in kayaking, much of the ability to execute the technique correctly is attributable to the athlete’s stability. However, with respect to the total score of the BESS test, significant negative correlations were observed between the SR and the CI at 200 and 500 m, only in the U14 category. The non-verification of this significant correlation in the U16 category again underscores the importance of years of practice in youth kayaking, as increased experience enhances boat stability, allowing a more efficient execution of the paddle stroke at higher SR.

Sitting height was significantly correlated with performance at 200 m in the U14 category. Interestingly, there was a moderate-to-high negative and significant correlation between the water/shoulder distance in both the 200 m and 500 m performances, suggesting that those who achieve the fastest times at 200 m and 500 m use a higher seat. It is an evidently complex situation; however, the results obtained regarding the SH–WSD difference and the SH:WSD ratio seem to corroborate the interpretation of the data. It was verified that athletes with the fastest times have lower SH:WSD ratios. This means that when sitting in their boat, the water/shoulder distance tends to approach the sitting height, so the difference between their sitting height and water/shoulder distance is smaller, indicating that the seat must be higher.

Even when the SH–WSD difference was lower in slower kayakers, the SH:WSD ratio was higher; this may be because these athletes had lower sitting height. This fact may confirm sitting height as a differentiating characteristic in kayaking and can be interpreted as an indicator that those who achieve the fastest times choose to use a higher seat, which may allow them to use longer paddles and, in turn, gain a mechanical advantage. Moreover, the higher the seat, the greater the imbalances, due to a higher center of mass relative to the water; this may also suggest that those with faster times will have greater technical proficiency, which may result from greater balance and stability. The moderate-to-high positive and significant correlation observed between the time at 200 and 500 m and the BESS test with foam surface seems to confirm this statement.

Considering the observed data on the SH:WSD ratio, it is possible to speculate that a normative value for this indicator differs between the two categories under study, U14 (1.77 ± 0.02 *n*) and U16 (1.64 ± 0.06 *n*). Regarding the PL:HGD ratio, the mean value obtained for the U14 (3.18 ± 0.28 *n*) and U16 (3.03 ± 0.18 *n*) categories is in line with Rademaker’s [42] suggestion that the correct placement of the hands on the shaft should divide the paddle into three equal lengths.

The physical fitness results underscore the need to develop optimally calibrated assessment batteries for race distances and age categories. For example, in the U14 group, the shuttle run test was significantly and negatively correlated with performance at 200 m and 500 m. In the U16 group, this significant negative correlation was observed for both pull-ups and sit-ups, possibly demonstrating a clear difference in strength between the age categories studied, especially if we consider the differences in the values recorded in the handgrip for U14 (27.11 ± 5.43 kg/f) and U16 (52.86 ± 7.49 kg/f). Handgrip strength is often used as a general indicator of overall muscle strength, as Wind et al. [43] demonstrated a strong correlation between grip strength and total muscle strength. Santos et al. (2011) [44] showed that handgrip strength correlated with the muscle mass indicator at the three maturity levels. A study by Rauch et al. (2002) [45], conducted with 315 children and adolescents (6 to 19 years), showed similarly higher handgrip strength with increasing age and observed a correlation between strength and stature. Furthermore, isometric strength increases with age in childhood and adolescence. At about 13 years of age, strength development in boys accelerates significantly, and longitudinal data show that adolescent increases in strength, motor performance, and absolute aerobic power occur in boys [46].

Regarding the paddle’s characteristics, an interesting finding is that in the U16 category, the blade width was significantly and positively correlated with time at 200 and 500 m. These data may indicate a choice for an oversized blade, with evident negative repercussions for performance, likely because it limits the kayaker’s ability to perform at a high SR and is not offset by increased SL. This observation is corroborated by Tsunokawa et al. (2019) [47], who found a decrease in SR when using hand paddles (which increase contact with the water) during swimming. But it differs from the evidence described in Sprigings et al. (2006) [48], which reports a method for personalizing blade size and indicates that the blade sizes used by most of the evaluated athletes were close to those predicted by the method. The apparent oversized blade observed in the present study may be due to the fact that, in many clubs, the high price of a new paddle often leaves young athletes with old paddles that are not suitable for them.

Previous studies have shown that higher SR is associated with better performance in elite adult kayakers [23,24]. The present study reports similar results across both categories, particularly for U16, with highly significant negative correlations between SR and time to complete the 200 and 500 m race, suggesting that higher SR is also associated with better performance in youth kayaking over both distances, more specifically, 119.33 ± 3.51 spm in the 200 m and 100.66 ± 2.30 spm in the 500 m for the U14 group, and 132.33 ± 2.51 spm in the 200 m and 109.66 ± 2.51 spm in the 500 m for the U16 group.

### Limitations and Practical Implications

This study is not without its limitations. The relatively small sample size and the exclusive inclusion of male Iberian kayakers limit the generalizability of the results. Secondly, the cross-sectional design prevents causal inferences regarding the identified determinants of performance. Although biological maturation was considered, its influence may not have been fully isolated from training experience, particularly when comparing athletes in the U14 and U16 categories. Furthermore, the proposed assessment batteries have not been externally validated and require confirmation in larger, independent and longitudinal cohorts. Thus, given the limitations of the present study, the proposed assessment batteries should be implemented as complementary tools rather than definitive screening instruments for young sprint kayakers. Coaches and practitioners are encouraged to use these batteries alongside regular technical, physiological and anthropometric monitoring, while considering the athlete’s biological maturation and training history when interpreting results. Since maturation may substantially influence performance-related variables, training prescription and talent identification should prioritize biological rather than chronological age whenever possible. Before these assessment batteries are adopted more widely, they should be externally validated in larger and more diverse populations, including female athletes and kayakers from different competitive levels and geographical regions. Future research should also employ longitudinal designs to determine how performance determinants evolve throughout adolescence and to establish causal relationships between maturation, training adaptations and sprint performance. Finally, intervention studies are warranted to evaluate whether training programs developed from these assessment batteries effectively improve performance over time.

## 5. Conclusions

In conclusion, this study provides novel insights into the determinants of sprint kayaking performance in young male athletes by identifying age-specific variables associated with 200 m and 500 m performance in the U14 and U16 categories. Among the variables examined, water–shoulder distance emerged as a consistent predictor across both race distances and age groups, highlighting the importance of technical positioning during paddling. In contrast, the combination of physical fitness and balance variables associated with performance differed between categories, suggesting that the determinants of sprint performance evolve throughout adolescence as a consequence of biological maturation, physical development and accumulated training experience. These findings support the development of age-specific assessment batteries that reflect the distinct performance profiles of U14 and U16 kayakers. For U14 athletes, water–shoulder distance, aerobic capacity (20 m shuttle run) and dynamic balance (BESS) appear to provide the most relevant indicators of performance, whereas for U16 athletes, water–shoulder distance, upper-body strength (pull-ups) and trunk muscular endurance (sit-ups) are more closely associated with sprint success. Furthermore, the results reinforce the importance of developing training programs that promote the ability to maintain efficient paddling mechanics while performing at high stroke rates, thereby optimizing performance without compromising technical execution. Collectively, these findings emphasize that performance assessment and training prescription in young sprint kayakers should be tailored to the athlete’s developmental stage rather than based solely on chronological age. Although further validation in larger, more diverse and longitudinal cohorts is warranted, the proposed assessment batteries provide a practical framework for coaches and practitioners to monitor performance-related characteristics and support evidence-based training and talent development in youth sprint kayaking.

## Figures and Tables

**Table 1:** Descriptive statistics (mean ± SD) of training, maturity, anthropometry, physical fitness, balance, and performance variables in U14 and U16 kayakers, their correlation (*r_s_*) with 200 and 500 m performance, and significant differences between age groups.

	U14 (*n* = 16)			U16 (*n* = 8)		
	Mean (±SD)	*r_s_* 200 m	*r_s_* 500 m	Mean (±SD)	*r_s_* 200 m	*r_s_* 500 m
Chronological Age (years)	12.67 ± 0.47 ^§^	−0.352	−0.318	15.57 ± 0.73	−0.619	−0.810 *
***Training***						
Years of Practice (years)	2.25 ± 1.00 ^§^	−0.705 **	−0.676 **	7.06 ± 1.39	−0.100	−0.200
Hours per week (hours)	3.46 ± 1.08 ^§^	−0.778 **	−0.625 **	13.75 ± 1.38	−0.378	−0.378
***Maturity***						
Maturity offset (years)	−0.98 ± 0.82 ^§^	−0.493	−0.400	1.94 ± 0.51	−0.071	−0.310
APHV (years)	13.65 ± 0.65	0.497 *	0.394	13.62 ± 0.50	−0.429	−0.667
PAH (cm)	181.65 ± 5.69	−0.056	−0.132	181.18 ± 7.30	0.024	−0.071
PAH (%)	86.69 ± 3.03 ^§^	−0.525 *	−0.485	96.87 ± 2.70	−0.190	−0.381
***Anthropometry***						
Body Mass (kg)	46.93 ± 8.73 ^§^	−0.355	−0.459	71.50 ± 6.10	0.405	0.357
Stretch Stature (cm)	157.49 ± 9.83 ^§^	−0.371	−0.400	174.90 ± 4.73	−0.119	−0.262
Sitting Height (cm)	81.79 ± 5.61 ^§^	−0.566 *	−0.475	91.95 ± 2.36	0.048	0.120
Arm Span (cm)	159.32 ± 10.33 ^§^	−0.354	−0.399	179.17 ± 6.32	−0.071	−0.214
Arm Length (cm)	27.93 ± 1.80 ^§^	0.070	−0.077	31.06 ± 1.63	0.120	−0.168
Forearm Length (cm)	23.09 ± 1.98 ^§^	−0.272	−0.334	25.85 ± 1.58	−0.262	−0.167
Hand Length (cm)	17.13 ± 1.25 ^§^	−0.518 *	−0.404	18.68 ± 0.64	0.060	−0.060
Relaxed Arm Girth (cm)	22.45 ± 2.49 ^§^	−0.080	−0.187	28.95 ± 2.24	0.262	0.357
Flexed Tensed Arm Girth (cm)	24.50 ± 2.82 ^§^	−0.019	−0.137	31.98 ± 2.02	0.286	0.476
Chest Girth (cm)	80.21 ± 5.16 ^§^	−0.473	−0.610 *	97.50 ± 4.01	−0.071	−0.024
Humerus Breadth (cm)	6.33 ± 0.48 ^§^	−0.145	−0.215	7.26 ± 0.30	−0.096	−0.192
Biacromial Diameter (cm)	34.23 ± 3.00 ^§^	−0.343	−0.416	38.92 ± 2.49	0.084	0.275
***Limb Volumes***						
Arm Volume (cm^3^)	1450.99 ± 331.23 ^§^	−0.159	−0.274	2338.59 ± 275.70	0.071	−0.024
Forearm Volume (cm^3^)	680.80 ± 151.07 ^§^	−0.213	−0.324	1247.47 ± 186.99	−0.071	−0.119
Upper Limb Volume (cm^3^)	2131.80 ± 473.02 ^§^	−0.172	−0.244	3586.06 ± 451.50	−0.024	−0.071
Thigh Volume (cm^3^)	5963.41 ± 1523.77 ^§^	0.035	−0.132	10,004.78 ± 1337.03	0.595	0.500
Leg Volume (cm^3^)	1369.97 ± 219.01 ^§^	−0.034	−0.265	2257.29 ± 320.42	0.190	0.143
Lower Limb Volume (cm^3^)	7333.39 ± 1715.58 ^§^	0.035	−0.132	12,262.07 ± 1570.20	0.500	0.405
***Body Composition***						
Sum of Six Skinfolds (mm)	83.78 ± 26.44	−0.091	−0.018	82.87 ± 15.43	0.167	0.357
Estimated Fat Mass (%)	18.34 ± 2.91	−0.044	0.070	17.97 ± 3.36	0.181	0.374
***Equipment***						
Seat/Footrest Distance (cm)	81.50 ± 6.62 ^§^	−0.112	−0.276	90.37 ± 3.24	0.563	0.371
Water/Shoulder Distance (cm)	45.31 ± 3.78 ^§^	−0.551 *	−0.732 **	54.41 ± 4.13	−0.771 *	−0.747 *
Paddle Length (cm)	196.30 ± 7.07 ^§^	−0.382	−0.293	209.92 ± 3.19	−0.024	−0.263
Blade Length (cm)	45.18 ± 1.14 ^§^	−0.071	0.016	49.43 ± 0.56	0.255	0.332
Blade Width (cm)	14.71 ± 0.60 ^§^	0.061	0.105	16.11 ± 0.30	0.847 **	0.773 **
Angle Between Blades (º)	57.36 ± 10.14	−0.297	−0.606 *	60.36 ± 7.08	−0.286	−0.143
Handgrip Distance (cm)	62.16 ± 7.02 ^§^	−0.265	−0.124	69.46 ± 4.30	−0.663	−0.590
***Physical Fitness***						
Push-ups (reps)	16.31 ± 5.71 ^§^	0.034	0.040	26.00 ± 8.91	−0.333	−0.476
Pull-ups (reps)	2.56 ± 2.27 ^§^	−0.390	0.004	11.13 ± 4.67	−0.831 *	−0.880 *
Sit-ups (reps)	42.63 ± 21.43 ^§^	0.134	0.276	74.13 ± 8.35	−0.846 *	−0.873 *
Handgrip Strength (Kg/f)	27.11± 5.43 ^§^	−0.452	−0.482	52.86 ± 7.49	0.214	0.143
Sit and Reach (cm)	1.21 ± 6.89 ^§^	−0.405	−0.474	10.43 ± 6.67	−0.262	−0.167
Overhead Throw (m)	5.24 ± 1.28 ^§^	−0.694 **	−0.409	9.07 ± 1.58	0.000	−0.286
Right Lateral Throw (m)	3.18 ± 0.73 ^§^	−0.505 *	−0.483	5.11 ± 1.14	0.120	−0.072
Left Lateral Throw (m)	2.97 ± 0.82 ^§^	−0.585 *	−0.225	5.22 ± 1.07	0.071	−0.119
Shuttle Run (courses)	43.43 ± 15.82 ^§^	−0.687 **	−0.741 **	79.00 ± 12.79	−0.265	−0.482
***Postural stability***						
Firm Surface Total (*n*)	4.75 ± 2.56 ^§^	0.397	0.398	2.88 ± 1.88	0.528	− 0.405
Foam Surface Total (*n*)	10.06 ± 4.26	0.664 **	0.836 **	9.75 ± 5.25	0.854 **	0.805 *
Test Total (*n*)	14.81 ± 6.29	0.668 **	0.756 **	12.63 ± 6.73	0.747 *	0.699
***Performance***						
200 m time (s)	59.93 ± 5.42 ^§^	−	−0.693 **	45.75 ± 2.80	−	−0.976 **
200 m SR (spm)	112.37 ± 7.43 ^§^	−0.585 *	−0.493	126.25 ± 6.81	−0.952 **	−0.905 **
200 m SL (m)	1.79 ± 0.11 ^§^	−0.711 **	−0.433	2.08 ± 0.05	−0.395	−0.479
CI 200 m (*n*)	6.05 ± 0.78 ^§^	−0.921 **	−0.650 **	9.14 ± 0.70	−0.905 **	−0.929 **
500 m time (s)	162.15 ± 12.98 ^§^	0.693 **	−	126.78 ± 4.80	−0.976 **	−
500 m SR (spm)	98.37 ± 3.70 ^§^	−0.616 *	−0.786 **	106.37 ± 5.47	−0.898 **	−0.874 *
500 m SL (m)	1.89 ± 5.87 ^§^	−0.558 *	−0.740 **	2.22 ± 0.05	0.500	0.381
CI 500 m (*n*)	5.87 ± 0.66 ^§^	−0.642 **	−0.859 **	8.80 ± 0.35	−0.500	−0.595

APHV: age at peak height velocity; PAH: predicted adult height; SR: stroke rate; SL: stroke length; CI: cycle index. * Correlation is significant at level *p* < 0.05. ** Correlation is significant at level *p* < 0.01; ^§^ Significant difference (*p* < 0.05) between U14 and U16.

**Table 2:** Correlation between CA, training, maturity, anthropometry, physical fitness, balance, and performance characteristics, and SR and CI at 200 and 500 m (*r_s_*).

	U14 (*n* = 16)				U16 (*n* = 8)			
	SR		CI		SR		CI	
	*r_s_* 200 m SR	*r_s_* 200 m CI	*r_s_* 500 m SR	*r_s_* 500 m CI	*r_s_* 200 m SR	*r_s_* 200 m CI	*r_s_* 500 m SR	*r_s_* 500 m CI
Chronological Age (years)	−0.021	0.374	0.153	0.379	0.548	0.643	0.503	0.667
***Training***								
Years of Practice (years)	0.267	0.796 **	0.787 **	0.147	0.150	0.200	0.050	−0.100
Hours per week (hours)	0.511 *	0.681 **	0.642 **	0.793 **	0.504	0.252	0.055	−0.378
***Maturity***								
Maturity offset (years)	−0.118	0.676 **	0.177	0.576 *	0.071	0.048	0.084	0.357
APHV (years)	−0.009	−0.650 **	−0.135	−0.638 **	0.452	0.333	0.407	0.500
PAH (cm)	−0.471	0.318	0.024	0.291	0.143	−0.095	0.228	−0.476
PAH (%)	−0.047	0.659 **	0.231	0.650 **	0.048	0.286	−0.132	0.667
***Anthropometry***								
Body Mass (kg)	−0.170	0.491	0.121	0.691 *	−0.452	−0.190	−0.563	−0.167
Stretch Stature (cm)	−0.192	0.597 *	0.253	0.526 *	0.143	0.119	0.228	0.095
Sitting Height (cm)	−0.022	0.751 **	0.256	0.661 **	−0.108	0.048	0.036	0.120
Arm Span (cm)	−0.197	0.546 *	0.162	0.588 *	−0.024	0.048	−0.060	0.286
Arm Length (cm)	−0.460	0.137	−0.229	0.258	−0.120	−0.120	−0.229	0.048
Forearm Length (cm)	−0.313	0.494	−0.209	0.389	0.048	0.381	0.060	0.381
Hand Length (cm)	−0.051	0.661 **	0.138	0.589 *	−0.012	0.120	−0.030	−0.156
Relaxed Arm Girth (cm)	−0.283	0.214	−0.045	0.423	−0.333	−0.048	−0.491	−0.167
Flexed Tensed Arm Girth (cm)	−0.336	0.171	−0.015	0.325	−0.381	−0.143	−0.347	−0.429
Chest Girth (cm)	−0.033	0.613 *	0.325	0.741 **	−0.024	0.357	−0.144	0.000
Humerus Breadth (cm)	−0.381	0.307	0.004	0.410	0.084	0.228	−0.127	0.012
Biacromial Diameter (cm)	−0.084	0.544 *	0.151	0.597 *	−0.216	−0.144	0.277	0.192
***Limb Volumes***								
Arm Volume (cm^3^)	−0.352	0.376	0.119	0.406	−0.143	0.143	−0.299	0.071
Forearm Volume (cm^3^)	−0.328	0.435	0.192	0.379	0.095	0.119	−0.060	−0.286
Upper Limb Volume (cm^3^)	−0.363	0.397	0.134	0.335	−0.024	0.190	−0.132	−0.095
Thigh Volume (cm^3^)	−0.516 *	0.147	−0.150	0.315	−0.595	−0.500	−0.719 *	−0.190
Leg Volume (cm^3^)	−0.459	0.229	0.046	0.412	−0.357	−0.095	−0.515	0.333
Lower Limb Volume (cm^3^)	−0.516 *	0.147	−0.150	0.315	−0.524	−0.381	−0.683	−0.143
***Body Composition***								
Sum of Six Skinfolds (mm)	−0.241	0.085	−0.085	0.056	−0.119	0.024	0.072	−0.762 *
Estimated Fat Mass (%)	−0.054	0.010	−0.012	0.093	−0.133	0.012	0.036	−0.759 *
***Equipment***								
Seat/Footrest Distance (cm)	−0.390	0.334	0.126	0.440	−0.419	−0.467	−0.428	−0.335
Water/Shoulder Distance (cm)	0.218	0.631 **	0.499 *	0.758 **	0.699	0.627	0.618	0.639
Paddle Length (cm)	−0.197	0.517 *	0.132	0.452	−0.048	−0.072	0.048	0.407
Blade Length (cm)	−0.256	0.076	0.100	−0.003	−0.511	−0.089	−0.475	0.332
Blade Width (cm)	−0.351	−0.014	−0.007	−0.063	−0.884 **	−0.724 *	−0.976 **	−0.295
Angle Between Blades (º)	0.361	0.343	0.614 *	0.284	0.452	0.143	0.383	−0.357
Handgrip Distance (cm)	−0.339	0.431	0.185	0.156	0.819 *	0.470	0.830 *	−0.229
***Physical Fitness***								
Push-ups (reps)	0.162	0.052	0.179	−0.221	0.548	0.452	0.527	0.548
Pull-ups (reps)	0.322	0.361	−0.039	0.055	0.868 **	0.747 *	0.812 *	0.578
Sit-ups (reps)	−0.044	0.024	−0.068	−0.308	0.791 *	0.709 *	0.851 **	0.546
Handgrip Strength (Kg/f)	0.055	0.489	0.254	0.615 *	−0.095	−0.190	−0.108	−0.286
Sit and Reach (cm)	−0.047	0.462	−0.019	0.725 **	0.238	0.000	0.108	0.381
Overhead Throw (m)	0.263	0.685 **	0.158	0.620 *	0.095	−0.095	0.048	0.071
Right Lateral Throw (m)	0.072	0.578 *	0.375	0.497 *	−0.204	−0.084	−0.422	0.299
Left Lateral Throw (m)	0.122	0.628 **	0.043	0.447	−0.143	0.000	−0.395	0.643
Shuttle Run (courses)	0.820 **	0.541	0.653 **	0.553 *	0.422	0.386	0.594	0.229
***Postural stability***								
Firm Surface Total (*n*)	0.103	−0.500 *	−0.267	−0.452	−0.540	−0.331	−0.655	−0.282
Foam Surface Total (*n*)	−0.307	−0.666 **	−0.713 **	−0.676 **	−0.732 *	−0.756 *	−0.700	0.146
Test Total (*n)*	−0.234	−0.667 **	−0.611 *	−0.683 **	−0.627	−0.627	−0.606	0.048
***Performance***								
200 m time (s)	−0.585 *	−0.921 **	−0.616 *	−0.642 **	−0.952 **	−0.905 **	−0.898 **	−0.500
200 m SR (spm)	−	0.399	0.414	0.365	−	0.786 *	−0.934 **	0.333
200 m SL (m)	0.082	0.908 **	0.513 *	0.397	0.204	0.743 *	0.265	0.323
CI 200 m (*n*)	0.399	−	0.606 *	0.612 *	0.786 *	−	0.766 *	0.524
500 m time (s)	−0.493	−0.650 **	−0.786 **	−0.859 **	−0.905 **	−0.929 **	−0.874 **	−0.595
500 m SR (spm)	0.414	0.606 *	−	0.424	−0.934 **	0.766 *	−	0.216
500 m SL (m)	−0.493	0.582 *	−0.786 **	0.958 **	−0.905 **	−0.874 **	−0.874 **	0.429
CI 500 m (*n*)	0.414	0.612 *	0.424	−	−0.934 **	0.524	0.216	−

APHV: age at peak height velocity; PAH: predicted adult height; SR: stroke rate; SL: stroke length; CI: cycle index. * Correlation is significant at level *p* < 0.05. ** Correlation is significant at level *p* < 0.01.

**Table 3:** Mean (±SD) for the PL:HGD and SH:WSD ratios.

	U14 (*n* = 16)	U16 (*n* = 8)
	Mean (±SD)	Mean (±SD)
**PL:HGD**	3.18 ± 0.28	3.03 ± 0.18
**SH:WSD**	1.81 ± 0.13 ^§^	1.69 ± 0.09

PL:HGD: paddle length:handgrip distance; SH:WSD: sitting height:water/shoulder distance. ^§^ Significant difference (*p* < 0.05) between U14 and U16.

**Table 4:** Mean (± SD) for the SH, the WSD, and the SH–WSD difference for U14 and U16.

	Mean (±SD)	Top 3 200 m	Top 3 500 m	Bottom 3 200 m	Bottom 3 500 m
U14 (*n* = 16)					
***Anthropometry***					
Sitting Height (cm)	81.79 ± 5.61	85.50 ± 7.05	83.37 ± 4.02	81.63 ± 4.67	76.50 ± 2.18
***Equipment***					
Water/Shoulder Distance (cm)	45.31 ± 3.78	49.07 ± 2.65	47.30 ± 1.56	46.20 ± 2.51	41.73 ± 2.21
***Difference***					
SH–WSD (cm)	36.48 ± 4.39	36.43 ± 4.41	36.07 ± 3.09	35.43 ± 2.30	34.77 ± 3.58
U16 (*n* = 8)					
***Anthropometry***					
Sitting Height (cm)	91.95 ± 2.36	92.07 ± 3.75	92.07 ± 3.75	91.30 ± 1.61	91.30 ± 1.61
***Equipment***					
Water/Shoulder Distance (cm)	54.41 ± 4.13	56.10 ± 4.53	56.10 ± 4.53	51.57 ± 3.21	51.57 ± 3.21
***Difference***					
SH–WSD (cm)	37.66 ± 2.76	35.97 ± 0.96	35.97 ± 0.96	39.63 ± 3.52	39.63 ± 3.52

SH: sitting height; WSD: water/shoulder distance.

## Data Availability

The original contributions presented in this study are included in the article. Further inquiries can be directed to the corresponding author.

## Abbreviations

The following abbreviations are used in this manuscript:

1RM	One-rep max
APAH	Age at predicted adult height
BESS	Balance Error Scoring System
CA	Chronological age
CI	Cycle index
PL:HGD	Paddle length to handgrip distance
PHV	Peak height growth velocity
PAH%	Percentage of predicted adult height
PAH	Predicted adult height
SH	Sitting height
SH:WSD	Sitting height to water/shoulder distance
SL	Stroke length
SR	Stroke rate
spm	Strokes per minute
WSD	Water/shoulder distance
